# Guided online treatment in routine mental health care: an observational study on uptake, drop-out and effects

**DOI:** 10.1186/1471-244X-13-43

**Published:** 2013-01-31

**Authors:** Robin Kenter, Lisanne Warmerdam, Christine Brouwer-Dudokdewit, Pim Cuijpers, Annemieke van Straten

**Affiliations:** 1 Department of Clinical Psychology, VU University, Amsterdam, The Netherlands; 2 De BOSgroep, Badhoevedorp, The Netherlands; 3 The EMGO institute for Health and Care research, VU University, Amsterdam, The Netherlands

**Keywords:** E-mental health, Implementation, Mental healthcare, Depression, Anxiety, Burnout

## Abstract

**Background:**

Due to limited resources patients in the Netherlands often have to wait for a minimum of six weeks after registration for mental health care to receive their first treatment session. Offering guided online treatment might be an effective solution to reduce waiting time and to increase patient outcomes at relatively low cost. In this study we report on uptake, drop-out and effects of online problem solving treatment that was implemented in a mental health center.

**Methods:**

We studied all 104 consecutive patients aged 18–65 years with elevated symptoms of depression, anxiety and/or burnout who registered at the center during the first six months after implementation. They were offered a five week guided online treatment. At baseline, five weeks and twelve weeks we measured depressive (BDI-II), anxiety (HADS-A) and burnout symptoms (MBI).

**Results:**

A total of 55 patients (53%) agreed to start with the online treatment. Patients who accepted the online treatment were more often female, younger and lower educated than those who refused. There were no baseline differences in clinical symptoms between the groups. There were large between group effect sizes after five weeks for online treatment for depression (*d* = 0.94) and anxiety (*d* = 1.07), but not for burnout (*d* = −.07). At twelve weeks, when both groups had started regular face-to-face treatments, we no longer found significant differences between the groups, except for anxiety (*d* = 0.69).

**Conclusion:**

The results of this study show that the majority of patients prefer online guided online treatment instead of waiting for face-to-face treatment. Furthermore, online PST increases speed of recovery and can therefore be offered as a first step of treatment in mental healthcare.

## Background

Depression and anxiety disorders are common mental disorders with a lifetime prevalence of 20% for depression, and 16% for anxiety disorders [1]. This results in loss of quality of life and substantial costs for the individual as well as for the society [2,3]. Various effective face-to-face treatments are available [4]. However, due to economic reasons there is a growing demand for shorter and less expensive treatments. Use of guided self-help treatments have been frequently suggested as one of the solutions. Self-help is defined as “a psychological treatment in which the patient takes home a standardized psychological treatment protocol and works through it independently” [5]. Guided self-help aims at helping patients to understand treatment techniques, to motivate patients and to responds to difficulties the patient may confront when working with self-help material. This might be provided by email, websites, or telephone [6]. Randomized controlled trials (RTC’s) have shown that guided self-help treatments are effective in reducing symptoms of depression and anxiety, regardless of the format in which it is offered (book or Internet) [7-9].

The most well-known example of integrating self-help in everyday mental health care is the Improving Access to Psychological Therapies (IAPT) initiative in the UK, which offers low to high-intensity treatments according to a stepped care model. First patients with mild to moderate anxiety and depression receive guided self-help, delivered by psychological wellbeing practitioners. After that, patients in need of more treatment have access to more intense psychological interventions. Results show good outcomes [10]. The two year data show large uncontrolled effect sizes for depression (1.07, 95% CI: 0.88-1.29) and for anxiety (1.04, 95% CI: 0.88-1.23) with reliable and clinically significant change in 55% of cases [11].

In sum, from RCT’s we know that Internet guided self-help is effective and IAPT shows that guided self-help can be implemented effectively in routine care. But we lack data on implementation of Internet based treatment in routine mental healthcare.

In this study we used data of a mental health institution that implemented Internet treatment in standard care and offered it during the waitlist period, which is the time between application and the first face-to-face session.

We report on (1) the uptake of the Internet treatment, which is defined as the percentage of patients who accept the offer of online treatment during waiting for face-to-face treatment and the percentage of drop-out, (2) the demographic and clinical profile of patients who prefer online treatment, and (3) the symptom reduction due to the online treatment compared to the symptom reduction in the group of patients who declined the offer and decided to wait for regular face-to-face sessions.

## Method

### Design

All eligible patients who registered for help at a mental health service were offered online treatment before the onset of face-to-face treatment. Participants who were willing to use the five week online treatment could start with the treatment the same day they signed up for the study. The other participants started with five weeks of waiting. They were asked by phone why they declined to offer of online treatment. After five weeks, both groups received the regular face-to-face treatment.

### Procedure

The internet course was not offered to every patient but only to those 18 years or older, with elevated depressive symptoms (scoring ≥14 on the BDI-II), anxiety symptoms (scoring > 8 on the HADS) and/or work-related stress (scoring ≥ 2.2 on high emotional exhaustion in combination with ≤ 2.2 depersonalization or ≤ 3.66 personal accomplishment on the MBI). It was not offered to patients with suicidal thoughts, without Internet access, or without sufficient understanding of the Dutch language. All new eligible patients of the mental health care center were informed about the Internet course and the study, by e-mail and/or by phone. All participating patients gave written informed consent to use the routine outcome monitoring data for this study. The enrollment took place between October 2010 and April 2011.

### Online treatment

The online treatment program that was used in this study was a five week Problem Solving Treatment (PST) [8,12]. It has proved to be effective in reducing symptoms of depression and anxiety [8,9]. Also, the generic character of the online PST makes it suitable to address different kinds of symptoms. This is useful because of the high prevalence of comorbidity in psychiatric disorders [3,13] and can therefore fit well into a stepped-care model in specialized or primary mental health care institutions [14].

The online treatment has three steps. First, participants have to write down things that really matter to them. Second, they have to describe their current problems and subdivide these into unimportant problems (i.e. unrelated to the things that matter to them), solvable problems, and unsolvable problems (e.g. the death of a loved one). For each category a different technique is suggested to solve or cope with the problems. The core of the program consists of solving the solvable problems. This is done in six steps: (1) writing a full description of the problem, (2) generating multiple solutions, (3) selecting the best solution, (4) making a plan for carrying out the solution, (5) actually carrying out the solution and (6) evaluating whether the solution has solved the problem. During the last step participants create a plan for the future in which they write down how they will try to achieve the things that really matter to them. The course takes five weeks and consists of one lesson a week. The website of the course contains instructions, exercises, examples of people applying the principles of PST. All participants received support in the form of feedback from a trained Master student in Clinical Psychology. The feedback was given within three working days by email and consisted of guiding the participants through the course and more personalized feedback (e.g. psycho education on how to cope with depression, anxiety and/or stress). The time spent on feedback was approximately 40 minutes per lesson.

### Instruments

All participants were asked to fill out the questionnaires at baseline, five weeks later (or directly after the online treatment) and twelve weeks after baseline. A week prior to an assessment point, participants received an email alert. The self report questionnaires were administered online.

### Depressive symptoms

Depressive symptoms were measured with the 21-item Beck Depression Inventory Second Edition (BDI-II) [15], a widely used multiple-choice self-report inventory which detects, assesses, and monitors changes in the severity of depressive symptoms as listed in the American Psychiatric Association’s Diagnostic and Statistical Manual of Mental Disorders Fourth Edition (DSM-IV; 1994). It consists of questions with answers being scored on a 0–3 scale, about how the person felt the last week. The total score varies between 0 and 63 with higher scores indicating more depression. Scores below 14 indicate absence of depressive symptoms. We used this cut-off score as an indication for recovery from depressive symptoms. The validity of the BDI-II has been tested in different populations [15]. In our study the Cronbach’s alpha was .86.

### Anxiety symptoms

For measuring anxiety symptoms the 7-item anxiety subscale of the Dutch version of the Hospital Anxiety and Depression Scale (HADS) was used [16]. Item-responses are on a 0 to 3 scale. The total score varies from 0 to 21, with higher scores indicating greater degrees of symptom severity. The cut-off score of 8 is an inclusion criterion for this study. The Dutch version of the HADS showed good homogeneity and reliability [16]. In our study the Cronbach’s alpha was .55 for the anxiety subscale.

### Burnout symptoms

The Dutch version of the Maslach Burnout Inventory (MBI) was used to measure work related stress. It consists of 15 items and assesses three components: emotional exhaustion (EE), depersonalisation (D), and personal accomplishment (C). The items are written in the form of statements about personal feelings of attitudes and are answered on 7-point scale (ranging from 0, “never” to 6 “very often”). Higher scores on the two subscales emotional exhausting and depersonalisation indicate more experienced work-related stress, while lower scores on personal accomplishment corresponds to higher degrees of work-related stress. A score >2.2 on the subscale emotional exhaustion in combination with a score >2.2 on the subscale depersonalisation or a score <3.66 on personal accomplishment is an indication for a burnout syndrome. Its validity and reliability has been tested in different populations [17,18]. In our study the Cronbach’s alphas for the subscales emotional exhausting, depersonalisation and personal accomplishment were .91, .85 and .87.

### Quality of life

We used the sixth item of the EuroQol questionnaire (EQ-5D) to assess quality of life [19]. Participants are asked to rate their current health from 0 (worst imaginable) to 100 (best imaginable).

### Statistical analysis

Statistical analyses were conducted using SPSS 18 for Windows. Baseline differences in demographics and clinical characteristics between the two groups were investigated using Chi-square and independent samples t-tests. We examined the effects between the two groups on the three outcome measures (depression, anxiety and work related stress) separately. First, we used ANOVA and follow-up t-tests to evaluate differences between the two groups for each assessment point. Second, we used independent samples t-tests to investigate the difference in mean scores between the two groups. Third, we calculated between group effect sizes (Cohen’s *d*) by subtracting the post-test mean score of the WL group from the post-test mean score of the PST group, divided by the pooled standard deviation. Effect sizes larger than *d =* 0.8 were considered to be large, *d* = 0.5–0.8 moderate, and *d* = 0.2–0.5 small [20]. Fourth, clinical significant change was determined with norms for the outcome measure. We calculated differences between the two groups in the percentage of patients who had recovered. We considered participants to have recovered when they scored <14 on the BDI-II, < 8 on the HADS-A and < 2.2 on emotional exhaustion in combination with a score < 2.2 on depersonalisation or > 3.66 on personal accomplishment on the MBI. Improvement was determined following the suggestions of Jacobson and Truax [21] calculating a reliable change index (RC).

All analyses were performed on the intention-to-treat sample. Pre-test data were available for all participants. Missing values of post-test non-responders were imputated by multiple regression analyses using available baseline data, demographics as well as data on baseline severity, from all participants.

### Trail registration

Ethical approval and registration was not required for this study according to Dutch law.

## Results

### Uptake of the course and drop-out

**Figure 1:**
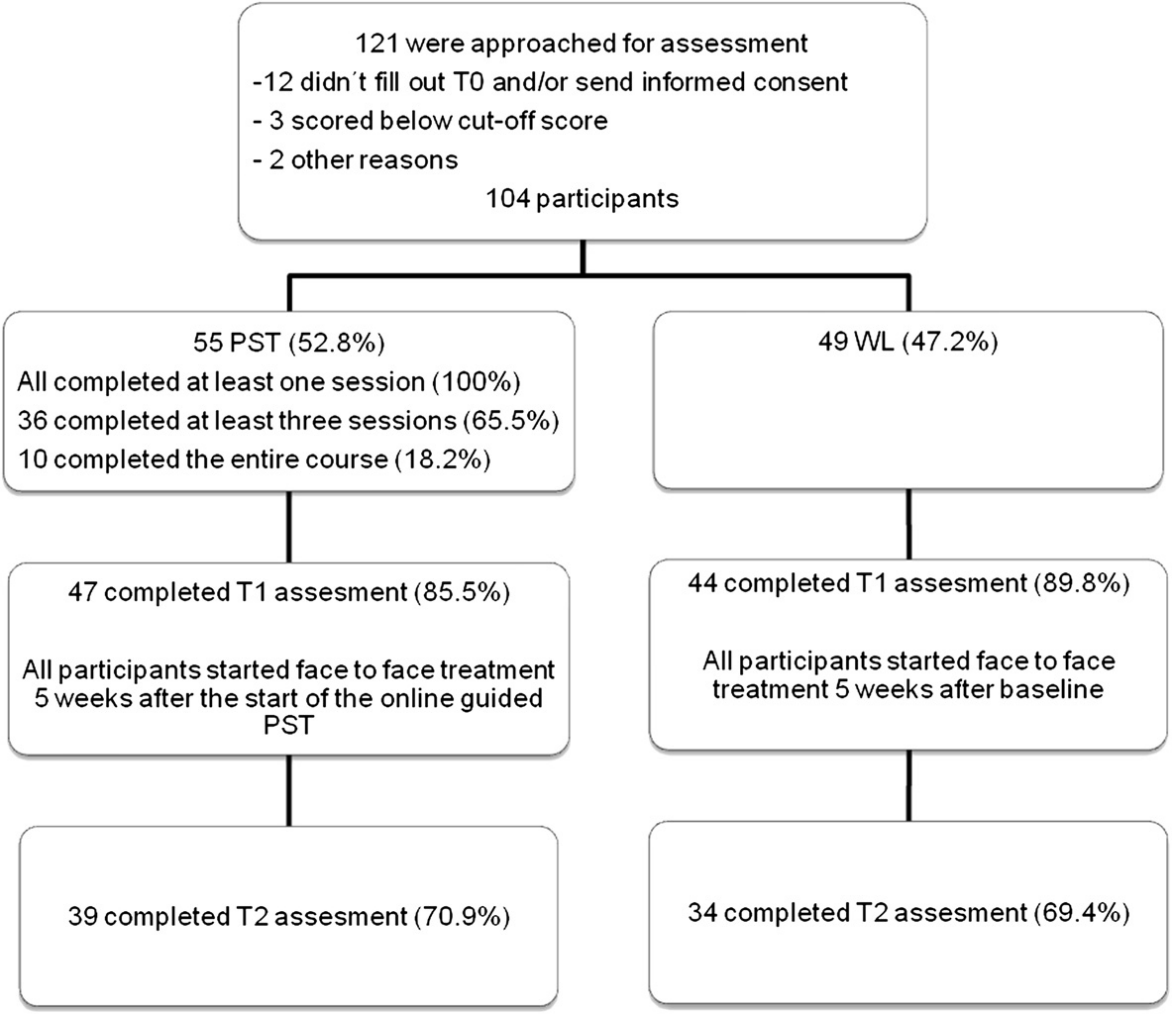
Participant flow.

Figure 1 shows the flow of participants. There were 121 patients interested in participating in this study. Of these patients, twelve did not fill out the baseline questionnaire or did not send back the informed consent, three patients scored below the cut-off score of 14 on the BDI-II or below 8 on the HADS-A and two patients didn’t participate for other reasons. This leaves 104 patients who participated in the study. Of those 104 participants, 55 (52.8%) chose to carry out the online PST, while the remaining 49 patients (47.2%) chose to wait for their face-to-face treatment (WL). All 55 participants who received online PST completed at least one session, 36 (65.5%) completed at least three sessions and 10 patients (18.2%) completed the entire course. Both the volunteers and the abstainers from PST received face to face therapy five weeks after admission.

At five weeks 12.5% (14.5% PST and 10.2% WL; *p* = .48) of the sample failed to return the study questionnaire, and this was 29.8% at twelve weeks (29.1% PST and 30.6% WL, *p* = .63). Reasons for dropout were unknown. Analyses show no significant differences in clinical or demographical characteristics between responders and non-responders.

### Description of the sample at baseline

Table 1 shows demographic characteristics. The total sample included 31 (29.8%) males and 73 (70.2%) females with a mean age of 37.6 years (*SD* = 10.8, 18–61). Of the 104 participants 51 (49.0%) completed intermediate vocational education or high school, 46 (44.2%) completed higher vocational education or university, and 7 (6.7%) completed primary education or lower general secondary education.

**Table 1:** Demographics at baseline

	**All (*N*** **= 104)**	**PST (*n*** **= 55)**	**WL (*n*** **= 49)**	**Statistic**
Age in years (*SD*)	37.6 (10.8)	33.7 (10.7)	42.1 (9.2)	*t*(102) = −4.81, *p* < .001
Female	73 (70.2)	48 (87.3)	25 (51.0)	*χ*^*2*^(1) = 16.27, *p* < .001
Education*				
-lower	7 (6.7)	4 (7.3)	3 (6.1)	*χ*^2^(2) = 8,62, *p* = .01
-middle	51 (49.0)	34 (61.8)	17 (34.7)	
-higher	46 (44.2)	17 (30.9)	29 (59.2)	

*Note*. Data are presented as n (%) of participants unless otherwise indicated.

*lower = primary education or lower general secondary education, middle = intermediate vocational education or high school, high = higher vocational education or university.

Participants in the online PST group were significant younger than those in the waiting list group (*t*(102) = −4.81, *p* < .001), were more frequently female (χ^2^(1) = 16.27, *p* < .001), and more frequently lower educated (χ^2^(2) = 8,62, *p* = .01). There were no significant baseline differences between the two groups regarding severity of depression (*t*(102) = −.817, *p* = .42), anxiety (*t*(102) = .713, *p* = .48), quality of life (*t*(102) = .773, *p* = .44), exhaustion (*t*(97) = −.101, *p* = .92), distance (*t*(97) = .015, *p* = .99) and competence (*t*(97) = −.382, *p* = .70) (see Table 2).

**Table 2:** Baseline differences and clinical outcomes after 5 weeks

	**Baseline**			**5 weeks**			***d *between**
	***M***	***SD***	***p***	***M***	***SD***	***p***	***(95% CI)***
*Depression*							
PST	23.5	7.0	.42	13.5	4.8	<.01	.94
WL	22.4	8.7		18.0	4.8		(.53-1.34)
*Anxiety*							
PST	10.6	2.9	.48	7.9	2.2	<.01	1.07
WL	11.1	2.4		10.3	2.3		(.66-1.48)
*QoL*							
PST	50.5	17.0	.44	68.9	7.8	<.01	.97
WL	47.9	17.7		58.0	14		(.57-1.34)
*Burnout EE*							
PST	2.5	1.7	.92	2.5	1.3	.84	-.06
WL	2.6	1.4		2.4	1.8		(−.46-.33)
*Burnout D*							
PST	2.0	1.5	.99	2.0	1.4	.79	-.07
WL	2.0	1.5		1.9	1.6		(−.46-.33)
*Burnout C*							
PST	3.5	1.3	.70	3.7	1.1	.78	-.08
WL	3.6	1.3		3.6	1.5		(−.32-.47)

### Clinical outcomes

The mean scores on depression, anxiety, burnout and quality of life were calculated for both groups at each assessment (see Table 2).

**Figure 2:**
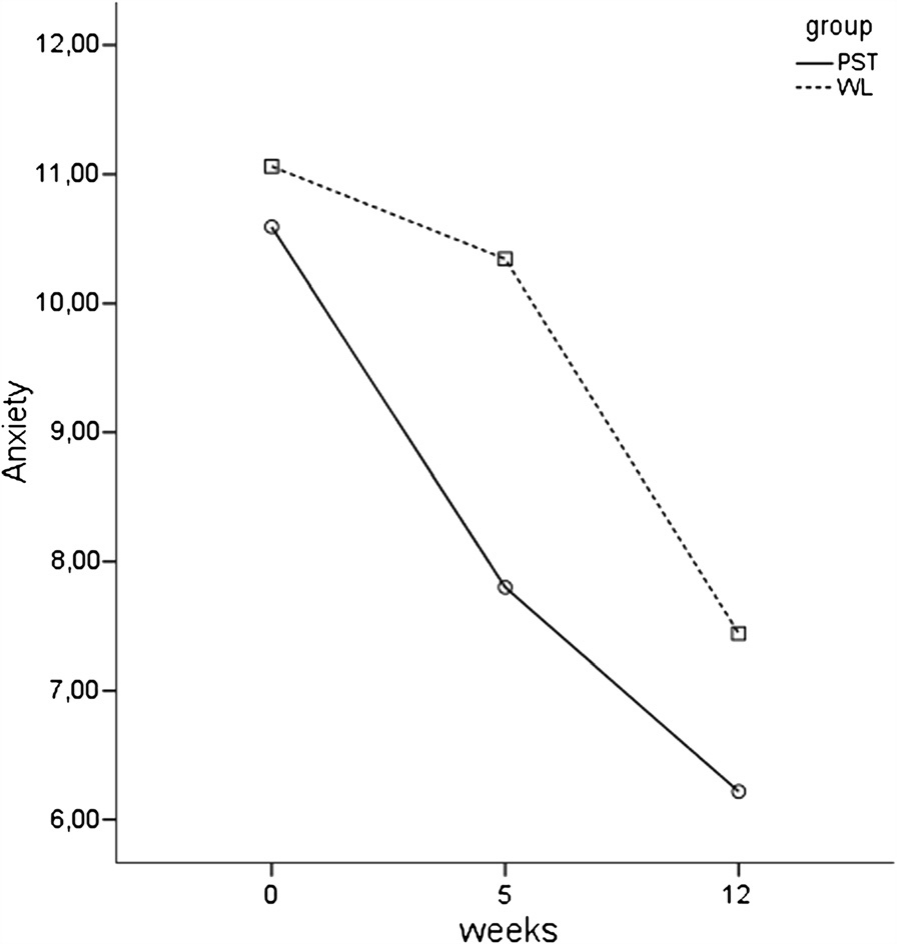
Improvement in anxiety.

A repeated measures ANOVA was conducted to evaluate the effect of online PST versus WL and time. There was significant overall improvement over time for both groups on anxiety scores, *F*(2, 33) = 76.90, *p* < .01. Results also showed a significant group x time interaction, *F*(2, 33) = 5.85, *p* = .01. Follow-up interaction comparisons were conducted to evaluate the differences between the two groups for each time period. Two pairwise comparisons were significant controlling for familywise error rates across the three tests at the .05 level using the Holm’s sequential Bonferroni procedure. There were significant differences in scores on anxiety between the two groups between baseline and five weeks (*t*(43) = −5.11, *p* < .01), and between five weeks and twelve weeks (*t*(33) = −2.55, *p* = .02). As Figure 2 shows, mean anxiety scores after five weeks were significant lower in PST than in WL, and after twelve weeks.

**Figure 3:**
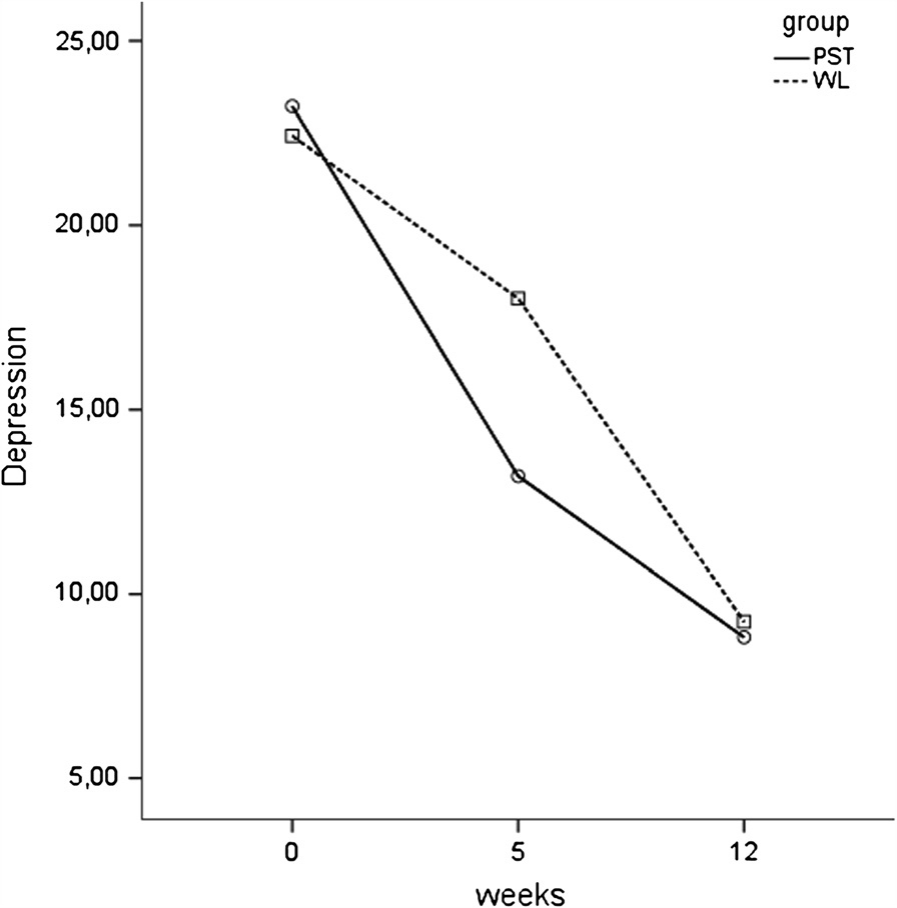
Improvement in depression.

Regarding depression scores, significant overall improvement over time was found for all groups on the BDI-II, *F*(2, 32) = 161.28, *p* < .01. Results also showed a significant group x time interaction on the depression scores, *F*(2, 32) = 16.46, *p* < .01. As Figure 3 shows, mean depression scores after five weeks were significant lower in PST than in WL, *t*(48) = −4.75, *p* < .01. The scores on depression did not significantly differ between the two groups after twelve weeks.

**Figure 4:**
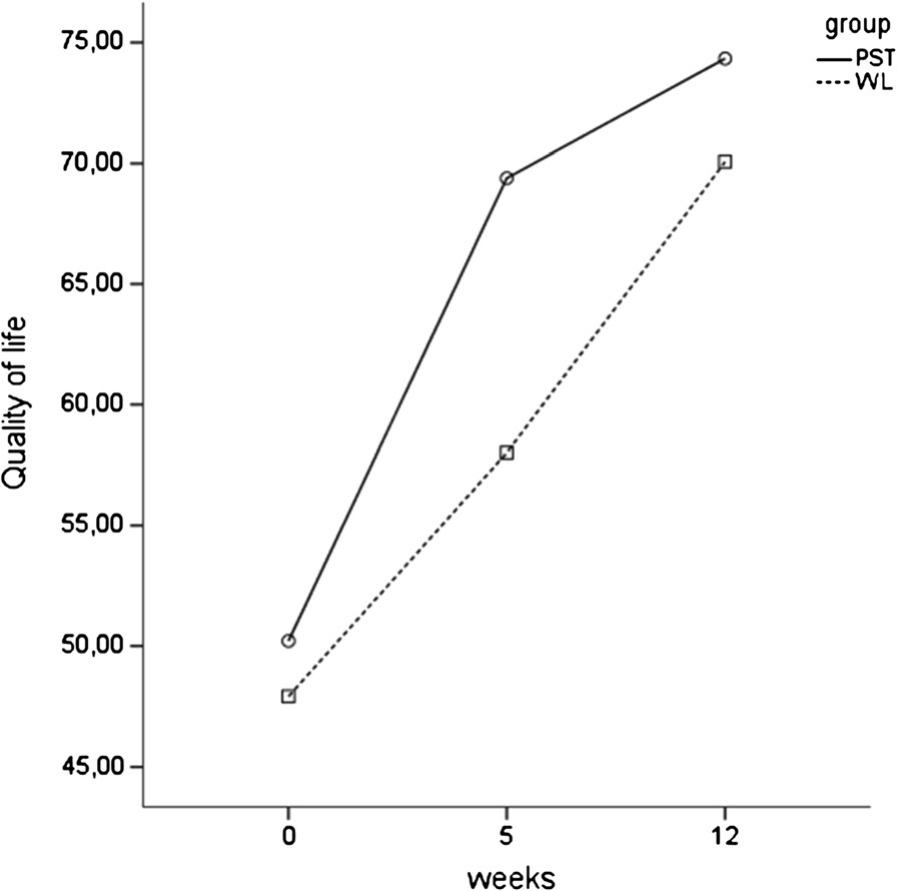
Improvement of quality of life.

As shown in Figure 4, there was significant overall improvement over time for all groups on quality of life, *F*(2, 32) = 113.77, *p* < .01. Furthermore, results showed a significant group x time interaction, *F*(2, 32) = 8.72, *p* < .01. Follow-up comparisons showed that the online PST group was significantly improved after five weeks compared to WL, *p* < .01. The mean quality of life scores after five weeks were significant higher in PST than in WL, *t*(48) = 4.91, *p* < .01. The scores on quality of life didn’t significantly differ after twelve weeks.

### Effect sizes

The five week between-group effect sizes were large for depression (*d* = .94, 95% CI:.53-1.34), anxiety (*d* = 1.07, 95% CI: .66-1.48) and quality of life (*d* = .97, 95% CI: .57-1.34) (Table 3). At twelve weeks, small effect sizes were found for depression (*d* = .11) and quality of life (*d* = .28), but anxiety still had a moderate effect size (*d* = .69).

**Table 3:** Clinical outcomes after 12 weeks

	**12 weeks**			***d *between**
	***M***	***SD***	***p***	
*Depression*				
PST	8.9	3.4	.63	.11
WL	9.3	3.6		
*Anxiety*				
PST	6.3	1.6	<.01	.69
WL	7.4	1.6		
*QoL*				
PST	73.3	10.3	.16	.28
WL	70.1	12.6		
*Burnout EE*				
PST	1.7	1.6	.38	.20
WL	1.4	1.4		
*Burnout D*				
PST	1.5	1.4	.27	.21
WL	1.2	1.4		
*Burnout C*				
PST	3.3	1.9	.14	-.37
WL	4.0	1.9		

### Clinically significant change

At baseline 17 participants in the PST group and 16 participants in the WL group fulfilled the criteria for burnout. Although there is a significant decrease in the number of people with burnout in the full sample (PST; *n =* 7, WL; *n =* 5), there are no significant differences between the two groups at five weeks (χ^2^(1) = .17, *p* = .68) and twelve weeks (χ^2^(1) = .39, *p* = .53).

At 5 weeks reliable improvement was 52.7% (*n* = 29) for depression, and 40% (*n* = 22) for anxiety for the PST group. For the waitlist group reliable improvement at 5 weeks was 20.4% (*n* = 10) for depression, and 14.3% (*n* = 7) for anxiety (depression: χ^2^ (1,104) = 11.55, *p* = .001; anxiety: χ^2^(1,104) = 8.52, *p* = .004). Reliable deterioration was 1% or less. Results show significant between-group differences in terms of clinically significant change on the BDI-II and HADS-A. Recovery (i.e. reliable improvement from a pretest score above cut-off to a post-test score below cut-off) occurred more often in the PST group (*n* = 28, 50.1%) after 5 weeks than in WL (*n* = 10, 20.4%) for depression (χ^2^(1,104) = 10.40, p = .001). This was also true for anxiety (PST; *n* = 10, 18.2%, WL; *n* = 3, 6.1%, χ^2^(1,104) = 3.45, *p* = .05).

The number of participants showing clinical significant change at 12 weeks for depression were *n* = 21 for PST and *n* = 17 for WL (χ^2^ (1,104) = .225, *p* = .635), and for anxiety *n* = 46 for PST and WL *n* = 29 (χ^2^ (1,104) = 7.705, *p* = .006).

Results are from analyses performed on intention to treat data. We also conducted the same analyses on the observed data from completers only. Results show no significant differences in comparison with intention to treat analyses.

## Discussion

### Main results

This study shows that Internet treatment in routine mental health care is feasible. About 50% of all patients preferred to carry out Internet treatment instead of waiting for face-to-face treatment. They were younger, more often female and more often lower educated than the patients who did not want to do this. Reasons patient chose to wait instead of carrying out the online treatment were the fear that personal information was not save on the Internet, the wish of talking to a person instead of a computer, and the believe of not having sufficient computer skills. The adherence to treatment was low, about two-thirds completed a substantial part of the online treatment (3 lessons or more), but only 18% carried it out completely. Several explanations for these outcomes can be suggested: it can be either due to characteristics of the patient sample, the way of treatment delivery, or the content of the online course itself which after three lessons doesn’t significantly differ from the previous lessons.

There were no significant differences between the two groups in clinical characteristics at baseline. Results show that patients who chose to carry out online PST improved more than patients who chose to stay on the waiting list. The online treatment had large between-group effect sizes after five weeks for depression, anxiety and quality of life. After five weeks both groups started with face-to-face treatment. The differences between the two groups then disappeared, although after twelve weeks the online PST group still outperformed the WL group for anxiety symptoms. Furthermore, 34% of the participants in the online PST group recovered to a clinically significant degree at five weeks while in the WL group only 9.1% had recovered after five weeks. Regarding burnout symptoms, online PST lead to a decrease in symptoms, but so did the control group. There were no significant differences between the two groups regarding burnout scores.

The online PST group showed rapid improvements after five weeks. Improvement directly after the start of treatment is not an unfamiliar finding [22]. Explanations for this effect could be due to non-specific factors like treatment expectations or perhaps the focus at the beginning of the online PST which consists of problem-solving [23]. In this study we found reductions of symptoms of depression, anxiety and burnout for the online PST group but also for the WL group. Perhaps the reduction of symptoms in the WL group was caused by test procedures (e.g. questionnaires) or spontaneous improvement. Improvement of the control group is common and also seen in other studies [24,25]. However, the outcomes of this study clearly show that participants who received online PST improved more.

Notable is the fact that despite the high recovery rates, all participants started face-to-face treatment after five weeks. Internet interventions are often considered as a first step in a stepped care model assuming that after this first step, not everybody needs a more intensive step, like face to face treatment. However, in this study the first face-to-face treatment session was already scheduled before start of the online treatment and the necessity of this appointment was not checked. We did not register the further uptake of treatment sessions.

### Limitations and future research

There are limitations of this study that should be noted. First, we only obtained data from one mental health care service. Second, we have no data available on why patients chose the online treatment over waiting. Perhaps these patients were more motivated and more ready to change. These factors, and also expectations of the PST intervention may be associated with the uptake and effects, instead of the intervention itself. Additional studies are required to examine patients motivation, and if the results hold for other mental health care institutions with patients from different ethnic backgrounds and socioeconomic groups.

Another limitation concerns the lack of diagnosis according to DSM-IV criteria. Online self-report was used to include participants. Even though we can assume that only patients with a DSM diagnoses were referred to the service by their GP, this is not certain. With regard to psychometric properties, the questionnaires used in this study were not tested for online use. Also, the HADS-A had a low alpha in our sample. The psychometric properties can be different from the paper versions but can also lead to more discloser of sensitive information in comparison with face-to-face interviews [26].

In this study, we were challenged with attrition and low adherence. Only 18% of the participants completed the whole online PST and 65% completed at least three sessions. Future research could focus on ways to minimize attrition and increase adherence, because adherence is correlated with outcome in cognitive therapy [27]. Also, we lack data on how many therapy sessions patients received after the five weeks of PST or waiting. Future research could evaluate the of cost effectiveness for the intervention and examine if patients require less face to face treatment after completing an Internet intervention.

## Conclusion

In sum, the results of this study show that the majority of patients prefer online guided treatment instead of waiting for face to face treatment. For symptoms of depression and anxiety online PST increases speed of recovery. Therefore, we recommend it to be used more broadly as a first step of treatment in mental health care.

## Competing interests

The authors declare that they have no competing interests.

## Authors’ contributions

RK carried out the study, participated in the design and coordination of the study, performed the collection and statistical analyses of data and wrote the manuscript. LW contributed with data analyses, the design of the study and the draft of the manuscript. CB participated in the coordination of the study and provided patients. PC and AvS supervised the study and critically revised the article for important intellectual content. All authors read and approved the final manuscript.

## Pre-publication history

The pre-publication history for this paper can be accessed here:

http://www.biomedcentral.com/1471-244X/13/43/prepub
